# Castor-oil biobased foam: the effect of the composition on the physical and mechanical properties *via* a statistical mixture design

**DOI:** 10.1039/d3su00374d

**Published:** 2024-01-24

**Authors:** Luiza Fernandes Soares, Júlio César dos Santos, Victor Augusto Araújo de Freitas, Robson Bruno Dutra Pereira, Tulio Hallak Panzera, Fabrizio Scarpa

**Affiliations:** a Centre for Innovation and Technology in Composite Materials – CIT^e^C, Department of Mechanical and Production Engineering, Federal University of São João del Rei-UFSJ Brazil fernandesluizasoares@gmail.com dsantosjcs@gmail.com robsondutra@ufsj.edu.br panzera@ufsj.edu.br; b Centre for Innovation and Technology in Composite Materials – CIT^e^C, Department of Natural Sciences, Federal University of São João del Rei-UFSJ Brazil victorfreitas@ufsj.edu.br; c Bristol Composites Institute, School of Civil, Aerospace and Design Engineering (CADE), University of Bristol University Walk BS8 1TR Bristol UK f.scarpa@bristol.ac.uk

## Abstract

PU foams are versatile materials that find applications in a wide range of products, from upholstery to packaging and construction. These foams consist primarily of two components, polyol and prepolymer, and their concentrations play a crucial role in determining their physical and mechanical properties. A second-order mixture design approach is used in this work to identify the significant components and their contributions on the physical–mechanical properties of biodegradable castor oil-based foams. The experimental design includes three components: two types of polyols and one prepolymer. These components are varied in nine distinct conditions to evaluate their effects on properties such as expansion rate, bulk density, compressive strength, and tensile strength. The Scheffé's quadratic model coefficients exhibit R-squared values higher than 0.84 in most cases. Chemical analysis using infrared spectroscopy confirms the successful formation of the urethane bond during the manufacturing process. The biobased foams developed in this work have densities ranging between 61 and 100 kg m^−3^, compressive modulus of 11–15 MPa and compressive strength between 273 and 429 kPa. The tensile modulus varies between 3.2 and 4.9 MPa, with a tensile strength in the range of 370–500 kPa. These results highlight the potential of biodegradable castor oil-based foams as promising alternative materials to traditional synthetic foams.

## Abstract 2

Optical microscope image of castor oil biobased foam.

## Graphical abstract

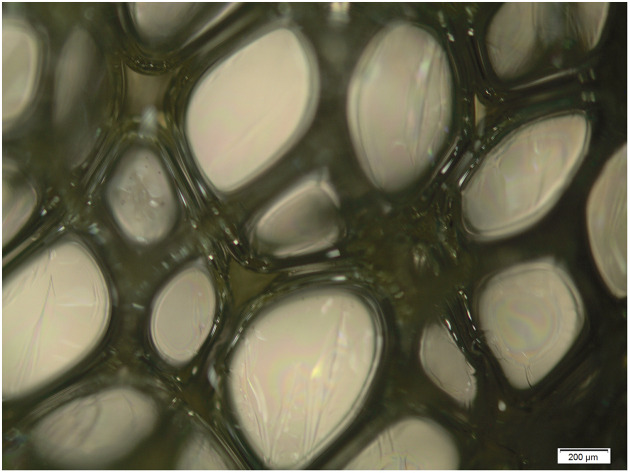

**Sustainability spotlight**

Polyurethane foams are extensively used in modern constructions, upholstery, packaging, and electronics. Polyurethane is traditionally obtained from petroleum derivatives, but recently biobased polyurethane has been produced from renewable and sustainable carbon plant resources. The paper describes a particular type of biobased polyurethane closed cell foam derived from castor oil, that is particularly low in toxicity, biodegradable, and relative low cost. Special emphasis is placed on a rigorous Design of Experiments that associates the mechanical performance of this biobased foam against the amounts of chemical compounds used in its fabrication. The Design of Experiments provides a map relating the manufacturing parameters for chemical/structural optimised configurations of the foams and contributes to future life cycle analysis for introducing castor oil closed cell foams into technical products.

## 1. Introduction

Polyurethane foams offer a wide range of applications, spanning from bedding and furniture to electronics and construction. Its market is expected to grow significantly at a rate of 7.5% between 2020 and 2025, reaching 54.3 billion dollars. Its versatility spans traditional and emerging markets, including countries such as India and Thailand. Additionally, its contribution to insulation of buildings with energy efficiency and unique properties drives global consumption.^1^

The first synthesis of polyurethane (PU) was made from petroleum derivatives. The current push towards the use of sustainable materials with a lower environmental impact has driven researchers to look for alternatives to fossil-based materials. Within this context, recycling or bio-based polymers have emerged as a sustainable alternative for engineering designs.^2^ Bio-based PUs have been successfully manufactured using renewable carbon resources, such as vegetable oils like castor, linseed, palm, olive, canola, corn, Karanja, and sunflower.^3^

Among the various vegetable oils, castor oil is considered the most significant due to its low toxicity, biodegradability, economic viability and easy accessibility.^4^ As a result, several works described in open literature make use of polyurethane foams derived from castor oil. More recent research focuses on incorporating additives to customise the material properties. Some recent research on castor oil foams includes the work of Silva and Oréfice,^5^ which incorporated cellulose–halloysite nanocomposite to adsorb multiple ions, such as nickel (Ni^2+^), cobalt (Co^2+^), and manganese (Mn^2+^). These ions are significant contaminants in certain Brazilian rivers due to mining dam failures. Zhang *et al.*^6^ examined flame retardant additives and their impact on the mechanical properties of the foams. Perera *et al.*^7^ functionalized the foam by modifying diatomaceous earth particles with octadecyl trichlorosilane, enabling an effective cleaning of oil spills in water. This foam has demonstrated a significant potential as a solution for addressing oil spills in aquatic environments.

The variation in the proportions of components in the synthesis of PUs leads to different physical and mechanical properties,^8^ making them suitable for various applications. While previous works on vegetable oil foams have primarily focused on the production of samples from laboratory-scale synthetised components, this study presents a novel approach by incorporating commercial raw materials using the mixture design method. By adopting this approach, the study aims to achieve two main objectives. The first is to investigate the individual effects of each component on the foam properties. The second is to assist users in selecting the optimal combination of those components to obtain the desired foam properties.

## 2. Materials and methods

### 2.1 Materials

The components of biobased castor oil are supplied by the Imperveg® (Brazil). The foam consists of two polyols (RD70 B and AGT 1315 B) and a prepolymer (RD70 A). These components are referred to as P1, P2 and PP, respectively. The foam is prepared by manually mixing polyols and prepolymers for about 1.5 minutes when the mixture begins to expand. The mixture is then poured into the medium-density fibreboard (MDF) mould (250 × 250 × 60 mm^3^) and cured for 14 days. Nine sample conditions are fabricated based on the simplex mixture design shown in Table 2. FTIR analysis is performed on the raw materials and foams.

### 2.2 Experimental planning

The original mixing problem arises due to the correlation between the components, wherein the sum of the proportions of each component must be equal to 1 
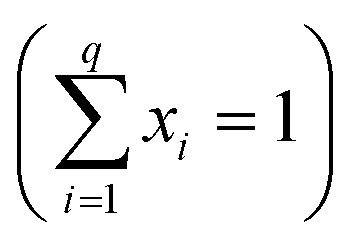
, where *q* is the number of components and *x*_*i*_ is the component proportions. As a result, the experimental region for mixture experiments is restricted, making traditional factorial or response surface designs inappropriate.^9^ Given the substantial number of combinations in the ternary system, it becomes possible to define a specific area for analysis.^10^

In this work, the R programming language is utilising for performing the experimental planning. The “mixexp” package^11^ is used to define the second-order planning. The “MixModel” function is used to fit a quadratic model for mixing experiments, referred to as the “Scheffé quadratic model”, as presented in eqn (1). In this equation, *y* represents the response variable, *β*_*i*_ represents the expected response at the vertex and the coefficients *β*_*ij*_ indicate the quadratic curvature along the edge of the simplex region. Additionally, this function also generates the correction coefficients, standard errors, and the corrected *R*^2^.^9^1
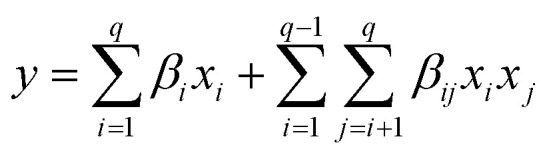


The simplex lattice mixture design is used to identify the effects of the three-phases compounding on the physical and mechanical properties of the foams. The final product must meet two prerequisites: (i) complete curing and (ii) the ability to cut the samples without compromising their physical integrity. Preliminary tests have been performed to establish the manufacturing requirements.

**Fig. 1:**
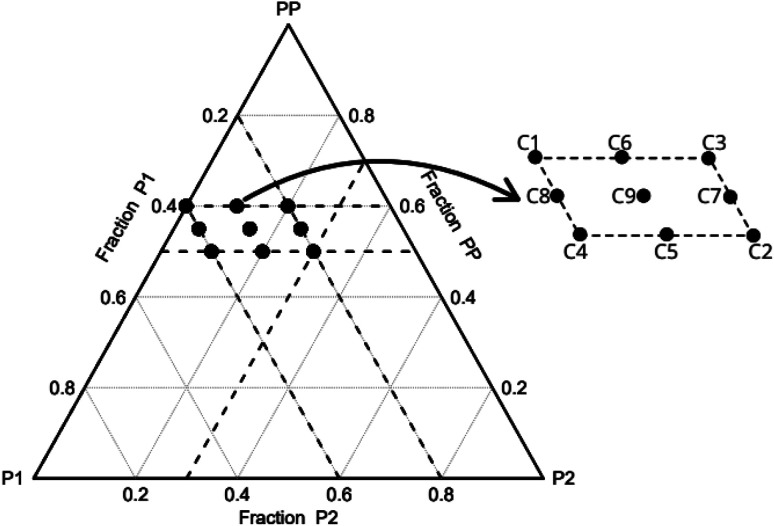
Simplex coordinates.

The incorporation of the component P2 was intended to increase the density and enhance the mechanical properties of the foams. To evaluate this effect, the initial concentration of P2 was set at 0 wt%. Additionally, preliminary tests have indicated that weight fractions of P2 in excess of 30% hinder the complete curing of the foams. It was observed that a PP concentration greater than 60 wt% leads to extremely fragile foams, making them unsuitable for cutting. In addition, if the concentration of P1 + P2 exceeds 50 wt%, the foam does not cure completely and/or exhibits a high contraction rate, which is also undesirable. Regarding the component P1, the manufacturer advises against going beyond 40% of its mass concentration. It is important however to include a minimum of 20% to ensure the mathematical completeness of the mixture's 100% mass composition. Consequently, the concentration range for each component PP, P1 and P2 is limited to 50–60, 20–40 and 0–30 wt%, respectively. Based on these limitations, the experimental planning consists of fourteen conditions (Table 1). Fig. 1 illustrates the experimental design and the simplex coordinates.

**Table 1:** Simplex lattice mixture design

Conditions	PP (wt%)	P1 (wt%)	P2 (wt%)
C1	0.60	0.40	0.00
C2	0.50	0.20	0.30
C3	0.60	0.20	0.20
C4	0.50	0.40	0.10
C5	0.50	0.30	0.20
C6	0.60	0.30	0.10
C7	0.55	0.20	0.25
C8	0.55	0.40	0.05
C9	0.55	0.30	0.15

### 2.3 Foam characterisation

The foams produced undergo chemical, physical, and mechanical characterisation. The chemical characterisation is performed using infrared spectroscopy (FT-IR). Spectra from liquid and solid samples are collected on a Shimadzu spectrometer equipped with the total attenuated reflection accessory (ATR), specifically the QATR10 model with a ZnSe crystal cell. The spectral range is configured from 4000 to 600 cm^−1^ with a resolution of 2 cm^−1^. For liquid samples, 10 μL is placed on the ATR crystal, while 10 mg is used for the solid samples.

**Fig. 2:**
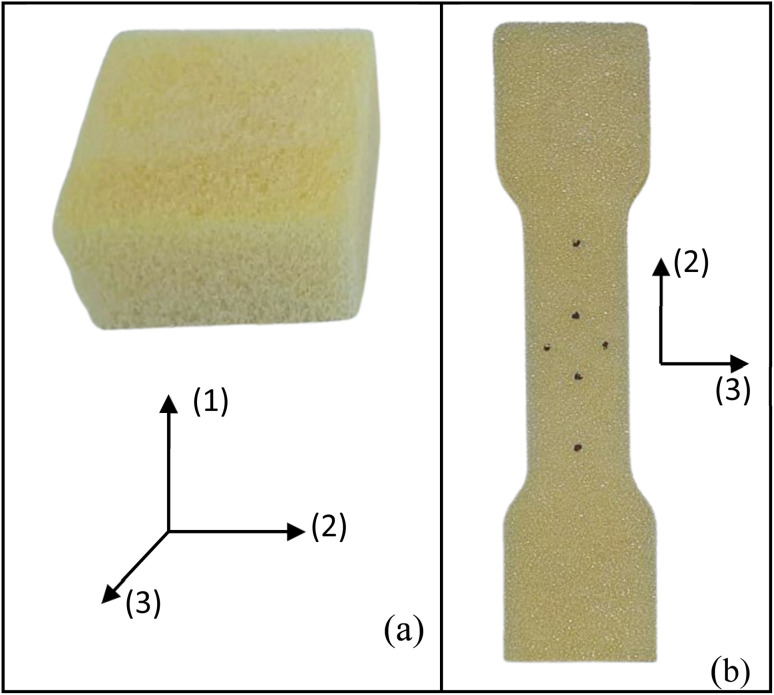
Compressive (a) and tensile (b) foam samples.

The physical and mechanical characterisations of the foams include the expansion rate, bulk density, compression, and tensile tests. The expansion rate is calculated by the ratio between the sample volume after curing and the initial mixture volume. Three samples are tested for each condition. Three measurements are taken for each condition using 20 g of foam poured into a 60 × 60 mm^2^ mould. The final volume is calculated by immersing the foam in a beaker filled with water. The bulk density is determined by calculating the ratio between mass and volume, following the guidelines outlined in ASTM D1622/D1622M.^12^ Four samples, measuring 30 × 50 × 50 mm^3^, are tested for each experimental condition. Foam specimens were precisely cut using a band saw to ensure accurate parallelism of the samples. Mass and volume measurements are obtained using a precision scale (0.001 g) and a digital caliper (0.001 mm). These samples are also used for the compressive testing (Fig. 2a).

The mechanical tests are performed using an Instron machine equipped with a 1 kN load cell. The mechanical testing has been carried out in a controlled environment at a temperature of 25 ± 2 °C and a humidity of 55 ± 3%. Four specimens are tested for each composition without any preload stage. The compression test follows the ASTM D1621-16 (ref. 13) protocols, with a test speed of 3 mm min^−1^. The test is performed along the rising direction of the foam, as shown by the axis (1) in Fig. 2a. Compressive modulus and strength are determined. The compressive modulus is determined by analysing the slope of the stress–strain curves in the linear regime within a stress range of 100–150 kPa. Additionally, specific properties are calculated by dividing the absolute values by their respective densities. The tensile test is carried out following the modified ISO 1926 standard,^14^ since samples of reduced size are used to fit into the jaws of the machine. A dog bone-shaped sample, measuring 7 × 37 × 150 mm^3^, is obtained by laser cutting horizontally the foam slices (plane 2–3, Fig. 2b), *i.e.*, along the direction perpendicular to the expansion of the foam (axis 1). A tensile test at a speed of 5 mm min^−1^ is considered. Tensile modulus and strength (absolute and specific) are then calculated.

## 3. Results

### 3.1 Physical characterisation

**Fig. 3:**
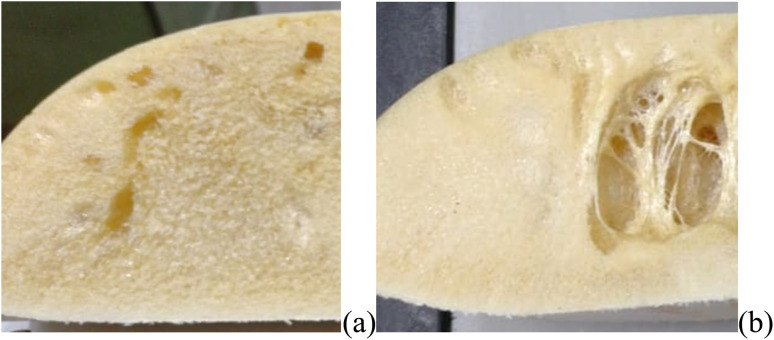
Manufacturing defects obtained for (a) C3 and (b) C6, C7 conditions.

Three experimental conditions (C3, C6 and C7) were not characterised since they experienced manufacturing defects. Condition C3 exhibited curing streak defects (Fig. 3a), while conditions C6 and C7 displayed cell collapse, as shown in Fig. 3b. These failures commonly attributed to slow healing processes or weakened cell walls.^15^

**Fig. 4:**
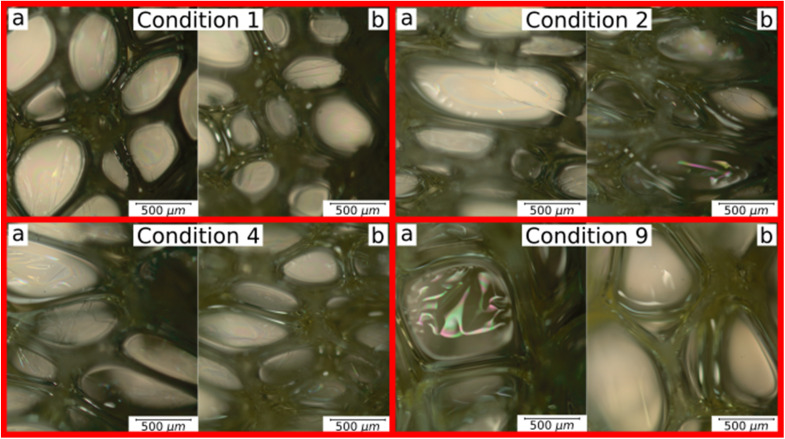
Microstructural analysis of C1, C2, C4 and C9 foam conditions: (a) plane 2–3 and (b) plane 1–3.

A microstructural analysis is performed on a set of foams (C1, C2, C4, and C9) taken from the vertex and central coordinates of the mixture design, as shown in Fig. 1. However, condition 3 has not been assessed due to manufacturing defects. The analysis is performed using an Olympus optical microscope with 5× magnification. The microscope is positioned both vertically (Fig. 4a) and laterally (Fig. 4b), allowing for the observation of cells in the 2–3 and 1–3 planes (Fig. 2a), respectively. The images reveal no significant physical variation between the foam directions. All foams are formed by rounded cells predominantly closed with a membrane. The cell sizes vary widely, ranging from 50 to 1000 μm, as depicted in Fig. 4.

**Fig. 5:**
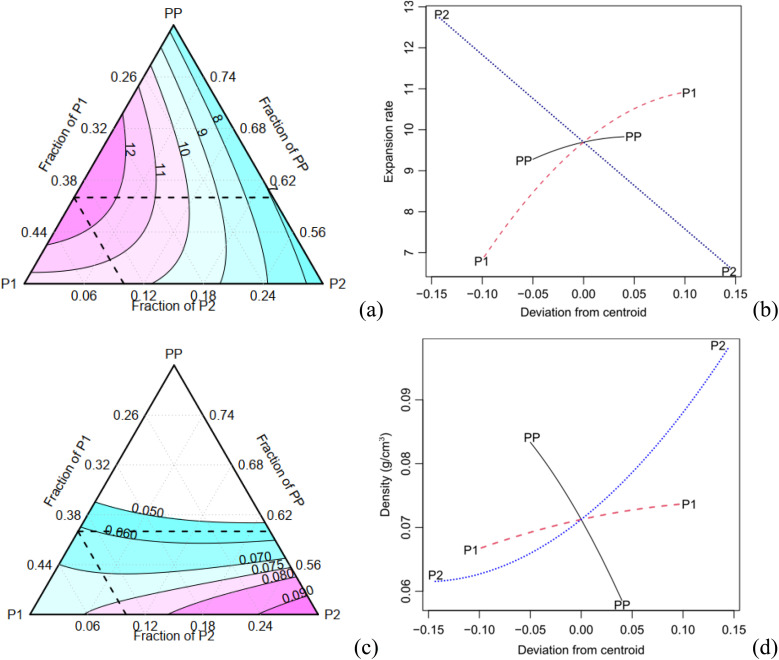
Contour and effect plots: (a, b) expansion rate and (c, d)bulk density.

The expansion rate and the values of the density, along with the corresponding Tukey test analysis, are presented in Table 2. Tukey tests statistically compare the means with a 95% confidence interval, where equivalent means are assigned the same letter group. Condition C1 exhibits the highest expansion rate (12.81), marked by the letter A, while condition C7 records the lowest value of 6.69, represented by the letter B. It is worth noting that certain conditions cannot be statistically distinguished from each other. For instance, conditions C2, C3, and C7 share the letter group E, while conditions C4, C6, and C9 belong to the letter group C. The quadratic model demonstrates a high adjusted-*R*^2^ value of 0.980. The Scheffé coefficients, provided in Table 4, reveal that the primary factor, P1, along with its interactions with PP and P2, are statistically significant at a confidence level of 0.05 (highlighted in bold in Table 3). This means that the factor P1 has a statistically significant impact on the experiment's responses. Fig. 5a illustrates the behaviour of the expansion rate in relation to component proportions, while Fig. 5b presents the corresponding effect graph. Both visual representations clearly illustrate that an increase in the P1 component leads to a greater expansion.

**Table 2:** Expansion rate and density data (mean ± standard deviation)

Condition	Expansion rate (%)	Tukey	Density (g cm^−3^)	Tukey
C1	12.81 ± 0.33	A	0.061 ± 0.002	E
C2	6.78 ± 0.23	E	0.100 ± 0.002	A
C3	7.03 ± 0.10	E	—	—
C4	10.31 ± 0.15	C	0.077 ± 0.001	C
C5	8.64 ± 0.04	D	0.086 ± 0.002	B
C6	10.12 ± 0.13	C	—	—
C7	6.69 ± 0.19	E	—	—
C8	11.64 ± 0.13	B	0.070 ± 0.001	D
C9	10.01 ± 0.07	C	0.071 ± 0.003	D

**Table 3:** Coefficients of the Scheffé quadratic model related to the expansion rate and the bulk density responses

*β*_*i*_ and *β*_*ij*_	Expansion rate		Density	
	Coefficients	Pr (>|*t*|)	Coefficients	Pr (>|*t*|)
PP	−11.395	0.337	−0.283	0.182
P1	−52.601	0.014*	−0.306	0.384
P2	6.860	0.694	0.421	0.008**
PP:P1	169.944	0.011*	1.475	0.188
PP:P2	2.889	0.959	0.300	0.632
P1:P2	54.389	0.001***	−0.275	0.091˙
Corrected *R*^2^	0.980		0.969	
Adjusted *R*^2^	0.999		0.999	
Signif. codes	0 ‘***’ 0.001 ‘**’ 0.01 ‘*’ 0.05 ‘˙’ 0.1 ‘ ’ 1			

The bulk density shows a variation of approximately 70% (Table 3). C2 has the highest density (0.1 g cm^−3^), indicated by the letter A, while C1 records the lowest values (0.061 g cm^−3^), marked with the letter E. Conditions C8 and C9 are considered statistically equivalent. The corrected *R*^2^ for the Scheffé quadratic density model is 0.969. The Scheffé coefficients are presented in Table 3. The main factor, P2, is significant at a confidence level of 0.01 (highlighted in bold in Table 3), and the interaction between P2 and P1 is significant at a confidence level of 0.1. Increasing the concentration of P2, which is the most statistically significant factor, leads to an increase in density. This behaviour can be observed in Fig. 5c and d, which present the contour and effect plots, respectively, for the mean density response.

### 3.2 Mechanical characterisation

#### 3.2.1 Compressive properties

**Fig. 6:**
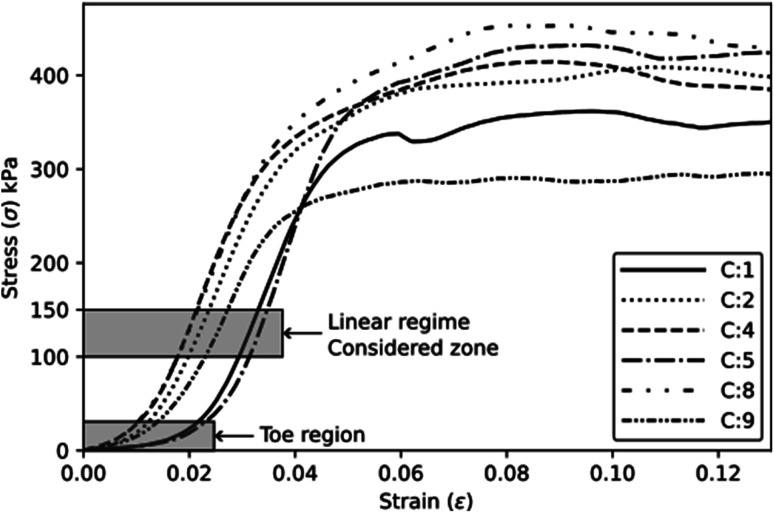
Typical compressive behaviour of the foams.

Fig. 6 shows some typical compressive stress *versus* strain curves obtained for the different experimental conditions. It is possible to observe a toe region corresponding to the contact between the plates and the specimen. Table 5 presents the mean absolute and specific compressive properties with their respective Tukey test analysis (*T*_K_). C5 achieves higher absolute elastic modulus and strength, while C9 leads to reduced properties. In terms of specific properties, C1 exhibits higher values, which can be attributed to its lower density, as indicated in Table 2.

The quadratic model for the absolute compressive properties achieves lower *R*^2^ values of 0.55 and 0.826 for the elastic modulus and compressive strength, respectively; as well as it provides corrected *R*^2^ values of 0.843 and 0.9 for their specific properties, respectively. The low *R*^2^ values can be attributed to the fact that most of the samples are statistically similar, as indicated by the Tukey test analysis. For example, in the case of absolute elastic modulus, only samples C5 and C9 exhibit statistical differences. Since the low *R*^2^ value obtained for the absolute compressive modulus, the discussion of Scheffé coefficients, as presented in Table 4, will be omitted. The contour and effect plots have been not generated here, as they do not provide an accurate approximation.

**Table 4:** Mean (absolute and specific) compressive properties Tukey test (*T*)

Condition	Absolute properties				Specific properties			
	Elastic modulus (MPa)	*T*_K_	Compressive strength (kPa)	*T*_K_	Specific modulus (MPa cm^3^ g^−1^)	*T*_K_	Specific compressive strength (kPa cm^3^ g^−1^)	*T*_K_
C1	13.49 ± 1.04	AB	381.75 ± 35.25	B	221.19 ± 16.99	A	6258.2 ± 577.87	A
C2	12.83 ± 0.70	AB	359.25 ± 26.75	AB	128.30 ± 7.00	C	3592.50 ± 267.50	C
C4	13.89 ± 1.04	AB	425.67 ± 9.56	AB	179.74 ± 13.51	B	5528.14 ± 124.10	AB
C5	15.04 ± 0.54	A	429.75 ± 33.25	AB	174.88 ± 6.34	B	4997.09 ± 386.63	B
C8	13.03 ± 0.92	AB	426.50 ± 48.25	A	186.11 ± 13.18	B	6092.86 ± 689.29	A
C9	11.11 ± 1.29	B	273.00 ± 16.50	C	156.41 ± 18.17	BC	3845.07 ± 232.29	C

**Fig. 7:**
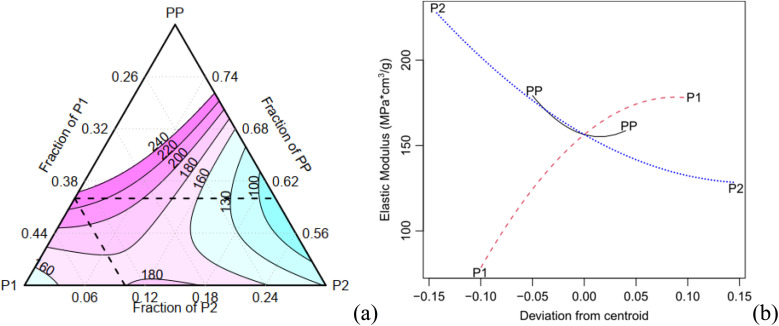
Contour plot for specific compressive properties (a) modulus (b) compressive strength.

Table 5 shows that the main factor P2 and its interaction with P1 are statistically significant for the specific compressive modulus, with a confidence level of 0.05. The contour and effect plots in Fig. 7 demonstrate that an increase of the component P2 results in a decrease of the elastic modulus.

**Table 5:** Scheffé coefficient, quadratic model for compressive properties

*β*_*i*_ and *β*_*ij*_	Compressive modulus				Compressive strength			
	Absolute		Specific		Absolute		Specific	
	Coefficients	Pr (>|*t*|)	Coefficients	Pr (>|*t*|)	Coefficients	Pr (>|*t*|)	Coefficients	Pr (>|*t*|)
PP	−77.36	0.479	781.34	0.512	−12212	0.001***	−137975	0.003**
P1	−173.93	0.352	−20.03	0.993	−18926	0.002**	−222962	0.004**
P2	201.47	0.013*	3041.87	0.004**	1209	0.559	2457	0.929
PP:P1	539.50	0.355	−951.17	0.893	63 663	0.001***	742 615	0.003**
PP:P2	−255.50	0.434	−8006.67	0.062˙	21 983	0.028*	263 685	0.045*
P1:P2	170.50	0.047*	2086.50	0.047*	3729	0.089˙	43 677	0.132
Corrected *R*^2^	0.550		0.843		0.826		0.900	
Signif. codes	0 ‘***’ 0.001 ‘**’ 0.01 ‘*’ 0.05 ‘˙’ 0.1 ‘ ’ 1							

In contrast, all components and their interactions are significant for the compressive strength at a confidence level of 0.05, except for P2 and its interaction with P1 (only for the specific property) as shown in Table 5.

**Fig. 8:**
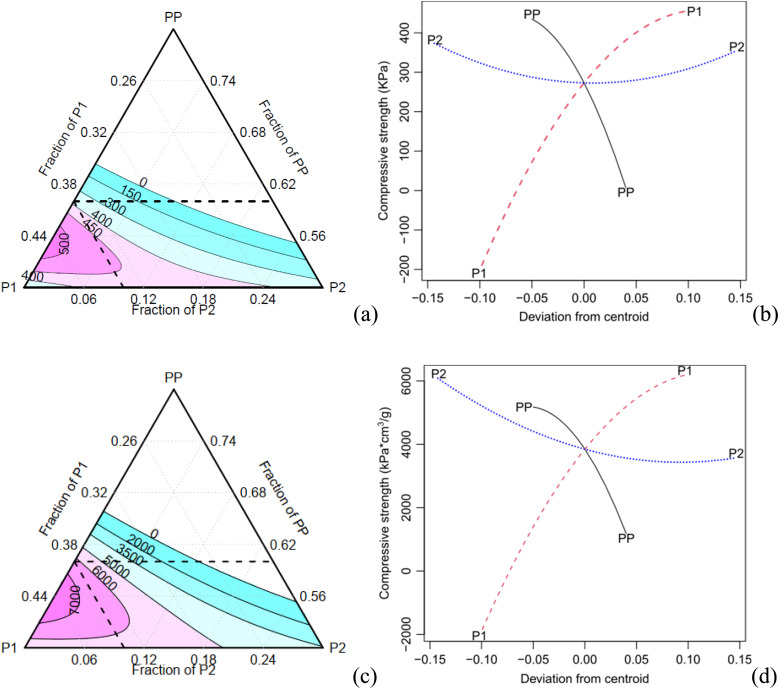
Contour and effect (plots: (a, b) absolute and(c, d) specific compressive strength.

The contour plot of the compressive strength exhibits a similar trend compared to the absolute and specific properties (Fig. 8). As the concentration of the component PP increases, both the specific modulus and compressive strength decrease. Conversely, an increase of the component P1 results in an increase in the specific compressive properties. When the component P2 increases, the density also increases, leading to a decrease in specific properties.

Table 6 presents a comparative analysis between the foam conditions of C5 and C8, and those made from different bio-oils, and two commonly foams used as the core material in sandwich panels for aerospace and defence applications. Samples C5 and C8 exhibit competitive properties when compared to the other bio-based foams.^1^ When comparing C8 foam, with a density of approximately 70 kg m^−3^, to Divinycell foam, which has a density of 60 kg m^−3^,^16^ the latter exhibits higher compressive strength while maintaining equivalent stiffness, demonstrating the promising application of castor-oil based foams.

**Table 6:** Comparative analysis between different (biobased and synthetic) PU foams

Foam	Density (kg m^−3^)	Compressive modulus (MPa)	Compressive strength (kPa)
C5 (castor-oil)	86	15	430
C8 (castor-oil)	70	13	426
Rapeseed-oil^1^	42	—	290
Soybean-oil^1^	46	—	400
Divinycell® U (minimum)^16^	80	21	1100
Divinycell® U (minimum)^16^	60	13	700

#### 3.2.2 Tensile properties

**Fig. 9:**
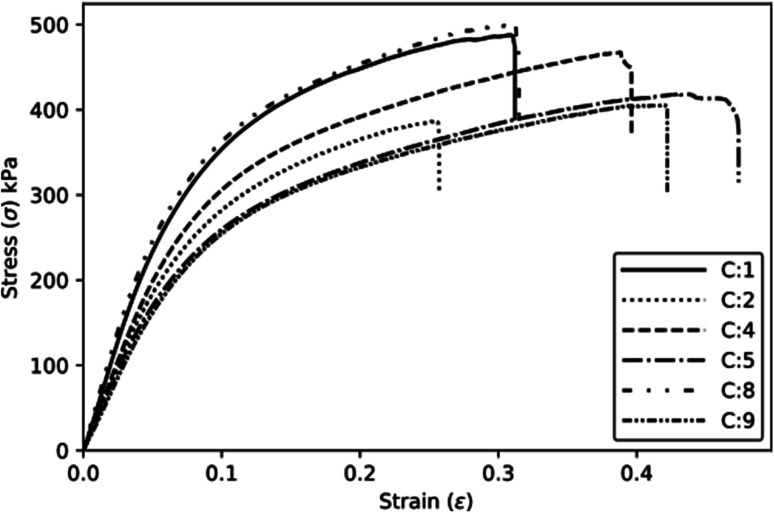
Typical tensile stress–strain curves.

Fig. 9 shows some typical behaviours of the foams during tensile loading. Table 7 presents the statistics related to the tensile data, as well as the results of the corresponding Tukey test (*T*). The mean stresses at failure possess a high value of the standard deviation, resulting in statistically equal means according to the Tukey test. The other properties also demonstrate minor variations among the means. The *R*^2^ values for the tensile properties are relatively low, with values of 0.84 and 0.778 for modulus and stress at failure, respectively. However, the specific tensile modulus and specific stress at failure achieve higher values of 0.925 and 0.956, respectively (Table 8).

**Table 7:** Mean (absolute and specific) tensile properties of the foams

Condition	Absolute properties				Specific properties			
	Young modulus (MPa)	*T*_K_	Stress at break (kPa)	*T*_K_	Specific Young modulus (MPa cm^3^ g^−1^)	*T*_K_	Specific stress at break (kPa cm^3^ g^−1^)	*T*_K_
C1	4.86 ± 0.25	AB	499.25 ± 18.75	A	79.59 ± 4.02	A	8184.43 ± 307.38	A
C2	3.96 ± 0.78	BC	374.25 ± 48.25	B	39.58 ± 7.83	B	3742.50 ± 482.50	E
C4	3.85 ± 0.17	CD	442.75 ± 15.75	AB	49.97 ± 2.24	B	5750.00 ± 204.55	C
C5	3.65 ± 0.15	CD	407.75 ± 14.75	B	42.47 ± 1.76	B	4741.28 ± 171.51	D
C8	5.66 ± 0.24	A	497.50 ± 6.50	A	80.89 ± 3.38	A	7107.14 ± 92.86	B
C9	3.16 ± 0.20	D	370.75 ± 17.13	B	44.47 ± 2.78	B	5221.83 ± 241.20	CD

**Table 8:** Coefficients of the Scheffé equation quadratic model for the absolute and specific tensile properties

*β*_*i*_ and *β*_*ij*_	Young modulus				Stress at break			
	Absolute		Specific		Absolute		Specific	
	Coefficients	Pr (>|*t*|)	Coefficients	Pr (>|*t*|)	Coefficients	Pr (>|*t*|)	Coefficients	Pr (>|*t*|)
PP	−184.16	**5 × 10^−^** ^**5**^ ***	−2250.4	0.0001***	−5195.8	0.064˙	−137975	0.003**
P1	−278.35	0.0001***	−3625.7	0.0002***	−8283.2	0.077^*.*^	−222962	0.004**
P2	−61.89	0.019*	−876.7	0.0139*	519.2	0.781	2457	0.929
PP:P1	944.54	**7 × 10^−^** ^**5**^ ***	12 000.4	0.0001***	28 875	0.052˙	742 615	0.003**
PP:P2	524.50	**9 × 10^−^** ^**5**^ ***	6444.5	0.0002***	10 600	0.198	263 685	0.045*
P1:P2	−41.96	0.131	−230.1	0.516	−75	0.970	43 677	0.132
Corrected *R*^2^	0.844		0.925		0.779		0.956	
Signif. codes	0 ‘***’ 0.001 ‘**’ 0.01 ‘*’ 0.05 ‘˙’ 0.1 ‘ ’ 1							

In the case of both the absolute and specific tensile moduli, all the Scheffé coefficients are found to be statistically significant at a confidence level of 0.05, except for the interaction P1:P2 (Table 8). The main factors PP, P1 and their interactions show significant effects on the response, with a confidence level of 0.1. As for the specific stress at break, all coefficients are found significant at a confidence level of 0.05, except for the main factor P2 and its interaction with P1.

**Fig. 10:**
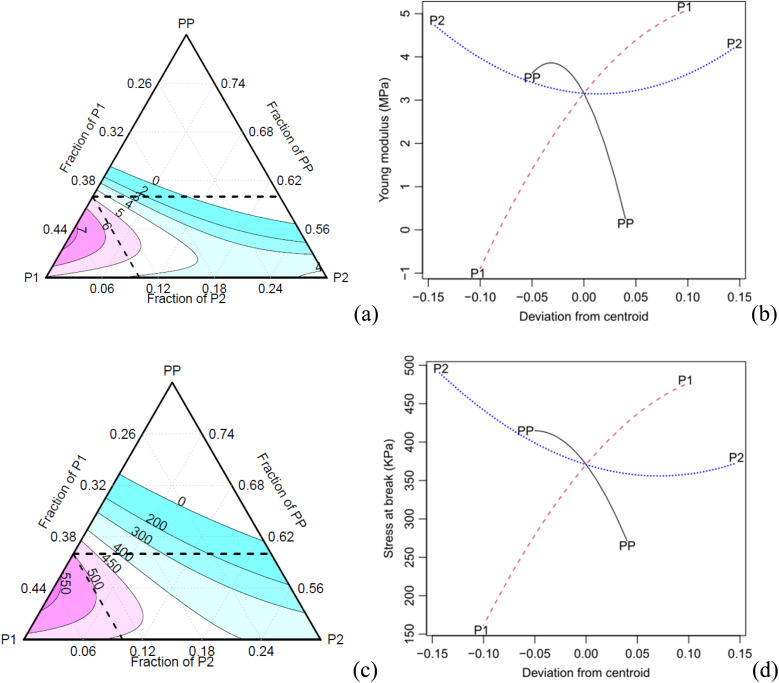
Contour effect plots: (a, b)tensile modulus and (c, d) stress at break.

The specific and absolute properties under tension show a trend like the one observed for the compressive properties (Fig. 10). The increase of the component P2 results in a decrease in specific properties due to the increase in density. The effect plots (Fig. 9b) show that both the PP and P2 components can be optimised, *i.e.*, the PP component exhibits a maximum point, while the P2 component has a minimum point for achieving an optimised modulus. Moreover, increasing the concentration of component P1 also contributed to an increase in the modulus. In terms of tensile strength, a similar trend is observed, although with a lower intensity.

### 3.3 Chemical analysis by FT-IR spectroscopy

#### 3.3.1 Polyurethane precursor

The polyol sample aligns very well with the standard castor oil spectrum profile, showing characteristic bands of ricinoleic acid, which is the primary fatty acid in castor oil. The broad and asymmetric vibration at approximately ∼3380 cm^−1^ indicates the presence of OH groups experiencing different neighbourhoods, including free hydroxyls, carboxylic acid hydroxyls and hydroxyls involved in hydrogen bonds. Additionally, the strong bands at 2922 and 2852 cm^−1^ are indicative of vibrations from CH_2_ and CH_3_ groups commonly found in aliphatic organic structures. The weak vibration around 3011 cm^−1^ is a distinguished feature of the C=C vibration observed in organic structures with a low degree of unsaturation (Fig. 8).^17,18^

**Fig. 11:**
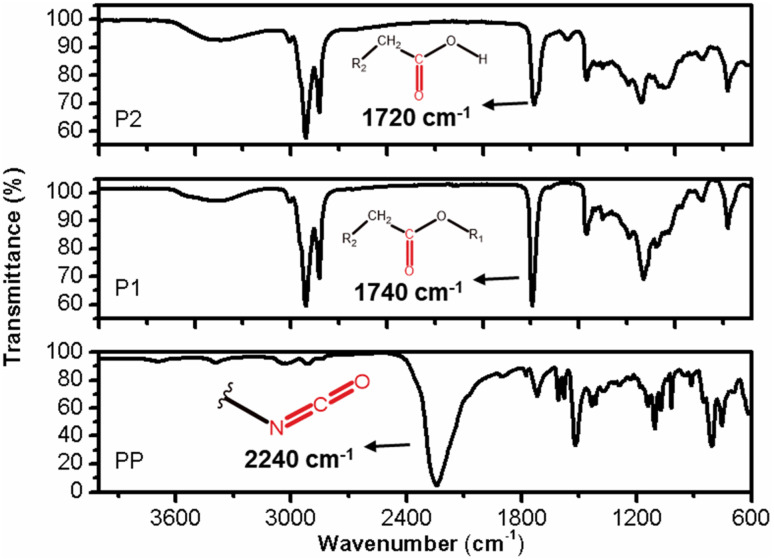
FT-IR spectra from the polyurethane precursors.

The FT-IR profiles of P1 and P2 exhibit a high degree of similarity, but subtle differences can be observed between them. In the case of sample P2, the vibration related to the C=O bond is both intense and symmetrical, occurring at approximately 1740 cm^−1^. Sample P1 has a maximum absorption shift at 1730 cm^−1^ and an inflexion point at 1708 cm^−1^_._ This discrepancy indicates that sample P2 is composed of structurally more uniform carbonyl groups, compared to those present in sample P1. Furthermore, the presence of a distinct set of vibrations around 1170 cm^−1^ in the two samples suggests that these carbonyls are associated with ester groups (R-COO-R), with varying carbon chain lengths in the case of sample P1 (Fig. 11).^17,19^

The IR profile of the PP samples exhibits typical characteristics of aromatic cyanates. A notable feature is the strong and asymmetric vibration occurring around 2237 cm^−1^, which corresponds to the stretching of the diisocyanate bond (–N=C=O). Additionally, the stretching vibration of the C=O bond can be observed at 1718 cm^−1^. The PP sample represents a diisocyanate with a short aliphatic carbon chain, as evidenced by the stretching vibrations of the CH_2_/CH_3_ bonds at 3038 and 2900 cm^−1^, respectively, as well as the corresponding deformation vibrations at 1430 and 1360 cm^−1^ (Fig. 9). The aromatic nature of this reagent is further confirmed by the simultaneous occurrence of vibrations at 803 and 752 cm^−1^, indicating the out-of-plane deformation of C–H bonds in aromatic compounds.^20^ Additionally, vibrations around 3040 cm^−1^ indicate the presence of sp^2^ carbon stretching, which is characteristics of aromatics (Fig. 11).^21^

#### 3.3.2 Polyurethane foams

**Fig. 12:**
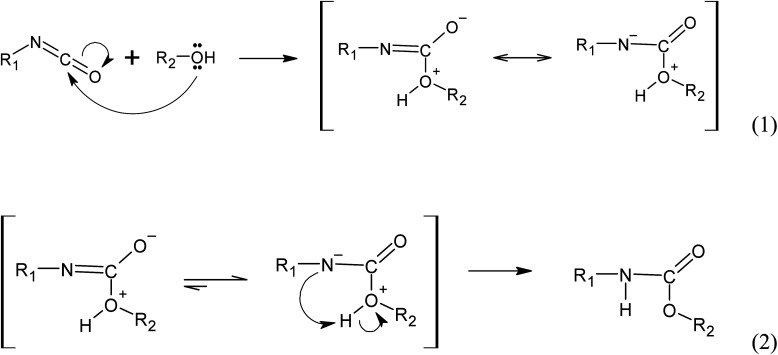
Simplified reaction mechanism of urethane bonds formation from the reaction between isocyanate group (R1-N=C=O) and an alcohol (R2-OH), where R1 and R2 are carbon side chains.

To facilitate a semiquantitative analysis of the chemical reaction progress during the formation of PU foams, all FT-IR spectra of the foams are subjected to normalization using a 15-point Savitzky–Golay smoothing method^22^ and a 10-point linear interpolation baseline correction. Notably, the vibration band associated with the stretching of the diisocyanate bond (N=C=O) at 2237 cm^−1^ is nearly absent in all samples. Only sample C2 exhibited a slightly stronger intensity of isocyanate vibration band compared to the other samples.^23^ The disappearance of this band (Fig. 12a) serves as evidence that nearly all the isocyanate has reacted with the hydroxyl groups of the polyol during the polymerisation process, resulting in the formation of urethane bonds as described in the following chemical reaction mechanism pathways.^20,23,24^

##### (i) Autocatalyzed reaction between polyols and isocyanate

The mechanism of the autocatalyzed reaction between polyols and isocyanate involves several steps. In the first step, the hydroxyl group of the polyols undergoes a nucleophilic attack on the carbon atom of the isocyanate group, leading to the formation of a zwitterionic transition state (reaction 1). This is followed by an intramolecular proton transfer, resulting in the formation of the urethane bond (reaction 2). Note that the specific details of the transition state and reaction mechanism may vary depending on the specific polyol and isocyanate used in the reaction. The cited source^24^ will provide more comprehensive information on this topic.

##### (ii) Water induced polyurethane bond formation

**Fig. 13:**
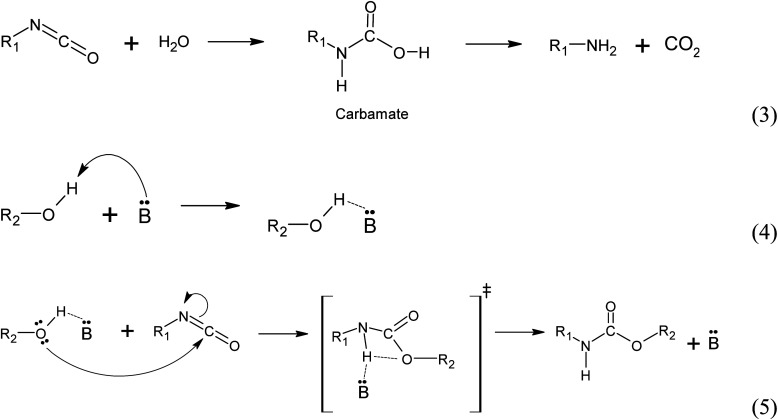
Simplified water catalysed reaction mechanism of urethane bonds formation from the reaction between isocyanate group (R1-N=C=O) and an alcohol (R2-OH), where R1 and R2 are carbon side chains, :B is the amine group.

The addition of water to the reaction mixture initiates a series of reactions with the isocyanate group. Firstly, water reacts with the isocyanate group to form a carbamate, which subsequently decomposes into an amine and CO_2_ (reaction 3). At the reaction conditions, the CO_2_ acts as a gas that promotes foam expansion. The resulting amine molecule, represented by the notation :B and denoted as R1-NH_2_, possesses chemical basic properties. As a basic catalyst, it coordinates with the hydrogen atom from the polyol hydroxyl group, forming an adduct (reaction 4). This adduct facilitates the nucleophilic attack from the polyol towards the carbon atom of the isocyanate group, resulting in the formation of a 4-ring transition state. The base catalyst (amine) stabilizes this transition state (reaction 5). Subsequently, the transition state rearranges to form the urethane bond and releases the amine (Fig. 13).^25^

**Fig. 14:**
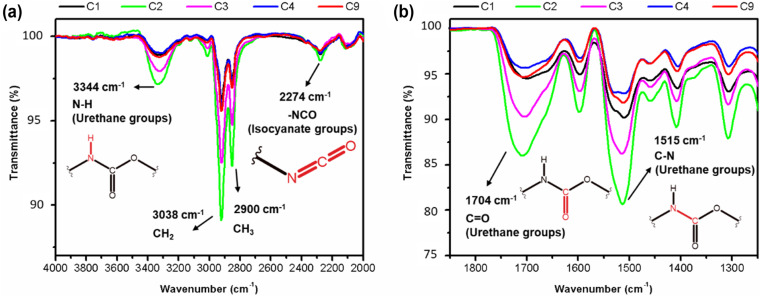
FT-IR spectra of from 4000–2000 cm^−1^ (a) and from 1900 to 1200 cm^−1^ (b) indicating the different extents of urethane linkages formation within the samples.

An increased symmetrical vibration around 3330 cm^−1^ is observed in all foam samples, indicating the presence of the amide group (–N–H). Additionally, a strong vibration at 1702 cm^−1^ corresponding to the stretching of the C=O bond and C–N bond, as well as a deformation at 1600 cm^−1^, are observed. These findings suggest successful formation of carbamates and –C–O–C ester groups, indicating the formation of urethane-type bonds in all samples. In addition, an asymmetric band and a shoulder band at 1702 cm^−1^ are observed in all samples, providing evidence of hydrogen bond formation between the polyurethane chains.^20,23^ Stretching and deformation vibrations related to the CH_2_ and CH_3_ groups present in the starting materials are observed at 2922 and 2852 cm^−1^ and 1460–1370 cm^−1^, respectively^20^ (Fig. 14a).

Subtle differences in the individual spectra of each PU foam however indicate that certain reaction conditions lead to a higher degree of urethane bond formation.^19^ It appears that the sequence of increasing urethane bond formation is as follows: C4 < C9 < C1 < C3 < C2. Therefore, sample C2 contains a higher content of urethane bonds, as evidenced by the greater intensities associated with the amide, carboxyl, carbamate, and ester bonds compared to the other samples (Fig. 14). With the formation of more urethane linkages in sample C2, an increase in hydrogen bond formation is also observed. These findings may explain why the sample C2 exhibits the highest density, as well as a low Young's modulus and low stress at break when compared to the other samples.

## 4. Conclusion

Biobased foams were produced using commercial raw materials and characterised accordingly. The foam architecture consisted of rounded closed cells ranging from 50 to 1000 μm. Out of nine assessed conditions, three exhibited manufacturing defects. The physical and mechanical properties of the foams were described using a second-order mixture design, which provided reasonably accurate approximations. In most cases, the *R*^2^ values exceeded 0.9, indicating a good fit of the Scheffé equation that describes each property. This suggests that one or more Scheffé equations can be used to optimise the foam properties for specific applications, such as achieving a combination of low density and high Young's modulus. Additionally, the significant components displayed substantial variations, particularly density, which could vary by up to 70%. Chemical analysis conducted using FT-IR confirmed the successful formation of the urethane bond. However, the extent of chemical linkages varied among the samples, which had a profound impact on the mechanical properties assessed in this study. An important consideration is the mixture proportion between moieties P1 and P2 in relation to the desired effective properties. Increasing the amount of P1 resulted in enhanced mechanical performance. The biobased foams exhibited densities ranging from 61 to 100 kg m^−3^, a compressive modulus of 11–15 MPa, and a compressive strength between 273 and 429 kPa. The tensile modulus ranged from 3.2 to 4.9 MPa, with a tensile strength in the range of 370–500 kPa. These properties show promise when compared to traditional synthetic foams. Future investigations will focus on analysing the fire retardant capabilities, acoustics, and damping features of these foams, offering potential avenues for further applications in packaging, sound management and energy absorption.

## Data availability

The raw/processed data required to reproduce these findings cannot be shared at this time as the data also forms part of an ongoing study.

## Conflicts of interest

The authors declare no conflicts of interest.
